# Body Measurements, Milk Composition and Productivity of Aruana Dromedary and Kazakh Bactrian Camel: The Basis for the Establishment of a National Standard

**DOI:** 10.3390/biology15080644

**Published:** 2026-04-19

**Authors:** Farida Amutova, Gaukhar Konuspayeva, Arailym Turgambek, Zhuldyz Baizhuma, Xenia Dronova, Assem Issayeva, Zauresh Bilal, Shynar Akhmetsadykova, Moldir Nurseitova, Nurlan Akhmetsadykov, Bernard Faye

**Affiliations:** 1 LLP “Scientific and Production Enterprise Antigen”, 4, Azerbayeva Str., Almaty 040905, Kazakhstan; amutovafb@gmail.com (F.A.); turgambekk.a@gmail.com (A.T.); bbaizhumazh@gmail.com (Z.B.); dronovksenia@gmail.com (X.D.); assem.good.1985@gmail.com (A.I.); bilalzauresh@gmail.com (Z.B.); mnurseitova2@gmail.com (M.N.); nurlan.akhmetsadykov@gmail.com (N.A.); 2 Biotechnology Department, Al-Farabi Kazakh National University, 71 Al-Farabi Avenue, Almaty 050040, Kazakhstan; 3 UMR SELMET, Center of International Cooperation on Agriculture Research for Development–CIRAD, Campus International de Baillarguet, 34398 Montpellier, France; bjfaye50@gmail.com; 4 LLP “Kazakh Research Institute for Livestock and Fodder Production”, Almaty 050035, Kazakhstan; shynar.akhmetsadykova@gmail.com

**Keywords:** *Aruana* dromedary, Bactrian camel, camel milk composition, pure breed, body measurements

## Abstract

Centuries of cohabitation between the two species of large camelids (dromedary and Bactrian) in Kazakhstan with a long practice of crossbreeding make it necessary to establish a national camel milk breed standard. To obtain data on purebred herds, the present study aimed to (i) describe the morphometric differences between the two species of large camelids based on some body and udder measurements, (ii) describe the composition of the milk (fat, proteins, minerals) of each species and determine the most discriminating parameters, (iii) verify the possible links between milk productivity and body measurements. The analyses, carried out on two “purebred” farms (respectively, 21 *Aruana* dromedary and 15 Bactrian camel of different parities but all sampled at the 5th month of lactation), made it possible to observe significant difference in the concentration of certain milk components (fat, respectively, 3.59 ± 1.22 vs. 5.27 ± 0.87%, protein: 3.18 ± 0.28 vs. 3.97 ± 0.29%, magnesium: 1357.07 ± 246.2 vs. 929.40 ± 166.8 µg/mL, manganese: 0.10 ± 0.05 vs. 0.03 ± 0.01 µg/mL). The combination of parameters (protein, Ca, Mg, Zn, Mn) made it possible to distinguish 97.6% of the animals. It was also possible to establish the lack of correlations between body measurements and milk production, except the link with heart girth (r = 0.529; *p* = 0.014) in dromedary only. By automatic classification, 3 different morphological types in dromedary camels and two in Bactrian camels, as well as two profiles of dromedary milk and 3 of Bactrian milk were identified. The present study provided preliminary results which should be validated on a larger number of farms with purebred animals in order to establish a national standard of camel milk, a necessary step for developing the market.

## Simple Summary

Camel breeding in Kazakhstan has two main types of camels: dromedary and Bactrian and until now no clear standards for camel milk based on pure breeds have been defined. This study aimed to better understand the differences between these two breeds of camels by examining their body characteristics, milk composition, and milk production. The research was conducted on two farms where animals are considered purebred. The results showed clear differences in several milk components, including fat and protein, and a combination of milk elements made it possible to distinguish almost all animals correctly. A relationship was also observed between body size and milk production. In addition, different body types and milk profiles were identified within each camel group. These findings provide important initial data that can support the development of standards for camel milk in Kazakhstan. However, further studies including more farms and animals are needed to confirm these results.

## 1. Introduction

Kazakhstan is one of the few countries in the world where the two species of large domestic camelids (one-humped dromedary—*Camelus dromedarius*—and double-humped Bactrian—*Camelus bactrianus*) are coexisting in significant numbers, often in the same regions and sometimes even within the same farm [1]. The dromedary in Kazakhstan belongs to a single breed named *Aruana*, started calling as Kazakh *Aruana*. This dromedary breed is in Kazakhstan at the northernmost part of its worldwide geographical distribution. It was introduced and selected for its dairy potential, leading to a specific dromedary Kazakh breed. At reverse, the Bactrian camel was present in the region for millennia and was the base of the nomad economy until the Bolshevik revolution [2]. Among these double-humped camels, different regional types were described [3], but they belong to the same genotype described as Kazakh Bactrian [3,4,5]. Both species are milk-producing animals, but with significant differences. *Aruana* camels generally exhibit higher milk yield, while Kazakh Bactrian camels produce milk with higher concentrations of fat and other solids [6,7]. In addition, the composition of milk varies, as in all dairy species, according to the lactation rank for both dromedary [6,8] and Bactrian [9].

In addition, with the increasing introduction of camel milk on national markets [10] and even the recent development of an international market for camel milk powder [11], the need to define standards has gradually become apparent to the players of the camel milk sector at the national and international levels. Recent initiatives under the aegis of the Codex Alimentarius have thus emerged to establish standards to support the development of the camel milk market. Several research studies have been carried out to determine the production conditions of organic milk [12] or the control of milk alteration of the product placed on the market [13]. However, the crossbreeding between dromedary and Bactrian camel is a common practice in Kazakhstan [14] leading to frequent mixture of genes between the two species. Now, to establish a national standard of camel milk by breed, it is necessary to collect data from pure breed. It was with the aim of having purebred farms that the State of Kazakhstan and, before it, the authorities of the Soviet Union put in place a control system of camel breeds in order to determine their status as “pure breed”. The control process is based on genealogical records, performance tests, and phenotypic observations of each animal at 2.5 and 5 years old. If all the animals meet the required standards, the farm receives certification from the Ministry of Agriculture attesting to its purebred status.

In the present study, the global objective of the paper was to determine the composition of the pure Kazakh *Aruana* and the pure Kazakh Bactrian milk according to their lactation rank and to their respective phenotype. In a second step, after describing the morphometric differences between the two species based on their body and udder measurements, and after investigating the links between milk productivity and body measurements, the objective will be to discuss how these findings may be useful for establishing a national standard in the context of Kazakhstan.

## 2. Materials and Methods

### 2.1. Animals and Farms

The study was conducted on two pure-breed camel farms located in two regions of Kazakhstan. The first farm was situated in the Atyrau region, north of the Caspian Sea, where purebred Kazakh Bactrian camels were bred. The second farm was located in the Turkistan region in southern Kazakhstan and maintained purebred camels of the Kazakh *Aruana* breed. Therefore, hybrids between dromedary and Bactrian camels were excluded from the study. In the first farm, 15 animals were selected at the 5th month of lactation. They were divided into one camel at the first lactation (Lact1), five camels at the second lactation (Lact2), four camels at the third lactation (Lact3), and five at lactation four or above (Lact4). In the second farm, twenty-one dromedaries were selected at the same lactation stage, and were divided into two Lact1, six Lact2, six Lact3 and seven Lact4.

All the animals in both farms were fed by natural fodders from the steppe without supplementation during the season of the study. The camel has a seasonal reproductive cycle with calving occurring in winter, and all the measurements and milk sampling being achieved at the 5th month of lactation. The diet corresponded to the natural steppic plants at the beginning of the summer season. At that moment of the year, the vegetation of the steppe was mainly composed of ephemeral vegetation at heading stage (*Leucanthemum vulgare*, *Stipa pennata* L., *Poa versicolor*, *Poa attenuata*), and drought-tolerant vegetation such as shrubs, subshrubs, diverse legume species, and halophytes (*Convolvulus arvensis*, *Alhagi maurorum*, *Elytrígia repens*, *Calamagrostis epigejos*, *Phalaris arundinacea*, *Caragana korshinskii*). Despite the distance between the two farms and their pastoral areas (around 1800 km), the floristic composition of the steppic vegetation was comparable [15].

### 2.2. Samples and Measurements

Milk volume was measured at two milking times (morning and evening) to get the dairy production for 24 h. Around 100 mL of milk sample was collected for each animal from the full day milk. Then the samples were put in an ice box and sent to the laboratory for analysis.

At the same time, body measurements were done on each sampled camel. The measurements included the head length (HL), the neck length (NL), the neck circumference (NC), the height at withers (HW), the heart girth (HG), the thigh circumference (TC), the teat length (TL), the udder width (UW), the mammary vein diameter (MWD), the foreleg length (FL), and the body length (BL) according to the methodology described in [16].

### 2.3. Laboratory Analyses

Regarding the milk composition, the following parameters were determined: Fat, protein, main minerals (Ca, K, Mg, P) and trace elements (Fe, Cu, Zn, Mn, Se).

The fat and protein were determined by using an automatic milk analyzer, Lactan (OOO VPK Sibagropribor ™, Novosibirsk, Russia). In addition, somatic cell count (SCC) was determined by using Somatos Mini (OOO VPK Sibagropribor™, Novosibirsk, Russia) and pH ST 10 (Ohaus, Parsippany, NJ, USA) was measured for detecting eventual hygienic failure that may have an effect on the gross and mineral composition.

The freezing point was also determined (FPD) by using the standard cryoscopic method with CryoStar-I cryoscopy (Funke Gerber™, Berlin, Germany) operating according to ISO 5764:2009 [17].

The elements were determined using an inductively coupled plasma optical emission spectrometer (ICP-OES) (Agilent 5800 ICP OES, Agilent Technologies, Santa Clara, CA, USA) equipped with a CCD Vista Chip III detector and a Peltier cooling system maintained at −40 °C. Sample preparation was carried out according to GOST 30538 97 with minor modifications: 50 ± 3 g of camel milk was mixed with 1 mL of nitric acid (1:1, *v*/*v*) and heated on a hot plate until charring was completed and smoke emission ceased [18]. The samples were then mineralized in an electric furnace starting at 250 °C, with the temperature gradually increased by 50 °C every 30 min to 500 °C over 10–15 h. The obtained white ash was dissolved in 1–2 mL of nitric acid (1:1, *v*/*v*) in the crucible and evaporated to moist salts, after which the residue was dissolved in 1% nitric acid and quantitatively transferred to a 25 mL volumetric flask. The ICP-OES system was operated under optimized conditions: RF power 1.20 kW, peristaltic pump speed 12 rpm, plasma gas flow rate 12 L min^−1^, nebulizer gas flow rate 0.70 L min^−1^, and radial plasma viewing mode. Calibration was performed using multi-element standard solutions prepared from certified reference materials, and analytical wavelengths were selected to minimize spectral interferences. The emission lines used for quantification were Ca (422.673 nm), K (766.491 nm), Mg (279.553 nm), P (213.618 nm), Cu (324.754 nm), Fe (248.300 nm), Mn (259.372 nm), Se (196.026 nm), Zn (202.548 nm).

### 2.4. Statistical Analyses

The following specific objectives were: (i) to detect the differences in milk composition between lactation rank and the species; (ii) to identify the most discriminating parameters between the two pure breeds; (iii) to identify the relationships between the milk composition, the milk productivity and the phenotypes within each species, determined by their body measurements, notably those regarding the udder measurements. (iv) to test the relationships between milk productivity, udder measurements, and milk components. To achieve those specific objectives, the statistical procedure was as follows:

- The between-species differences were tested by ANOVA after homogenization of the variance: the normality of the data was tested by the test of Shapiro–Wilk [19], and in case of heteroskedasticity, the variable was normalized by using the Newey–West adjusted method [20]. Due to an insufficient number of samples per lactation rank within species, the between-lactation differences were tested by a non-parametric Kruskal–Wallis test [21].
- The Discriminant Factorial Analysis (DFA) was applied to the data on milk composition, the explaining factor being the breed; the ascending stepwise model was used for determining the most discriminating parameters between the species and to assess the percentage of well-classed animals [22]. This percentage is obtained after cross-validation in the confusion matrix between a priori groups (species) and a posteriori groups (determined by the values of the milk components along the discriminant function).
- Multivariate analyses—Principal Components Analysis (PCA) was applied on the different data tables (milk composition, body measurements) for analyzing the relationships between all the variables. In addition, a cluster analysis by Automatic Hierarchical Classification (AHC) on the main factors of the PCA was applied to the different data tables; the obtained classes were described as types of milk composition and types of phenotypes, and their relationships were tested in a confusion matrix by a Chi^2^ test. The stability of the clusters was tested by iteration (*n* = 500).
- The correlation of Pearson was applied to assess the different relationships between the milk productivity and the variables describing the udder measurements, the phenotypes and the milk components.

For all these analyses, the software XLstat-2025.2.0 (Addinsoft, 2026 ©, Paris, France) was used.

## 3. Results

### 3.1. Interspecies and Lactational Differences

Globally, four parameters were significantly different between Bactrian and dromedary milk. On average, Bactrian milk was fattier, contained higher concentrations of protein but less magnesium and manganese (Table 1).

Regarding the lactation effect on the different parameters within each species, assessed by a non-parametric test, no significant difference was observed. For easier reading, only means were reported (Table 2).

### 3.2. Main Discriminating Parameters Between Species

According to the ascending stepwise model, the most discriminating parameter between Bactrian and dromedary milk was the protein concentration, followed by manganese, zinc, magnesium and calcium contents (Table 3). By adding another parameter (selenium), the probability (test F) was above 5%.

By retaining these 5 discriminating parameters, the percentage of well classed was 97.6%. Indeed, one dromedary had a milk profile similar to a Bactrian, leading to a posteriori misclassification. After iterative validation on a subsample of 10 animals, the percentage of well-classed animals appeared stable with 100% Bactrian well-classed and 95.2% dromedary well-classed.

### 3.3. Relationships Between Species and Milk Components

**Figure 1:**
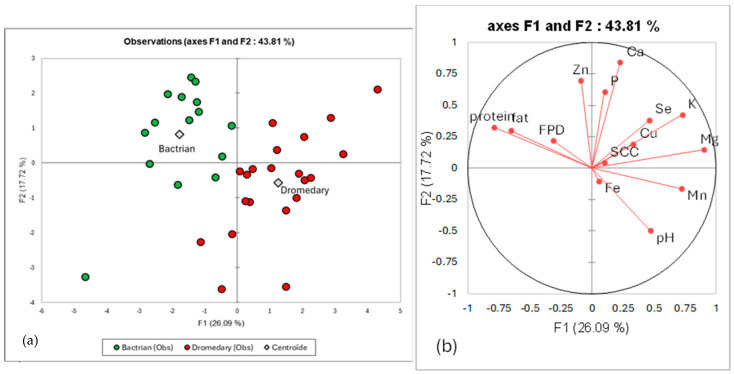
(**a**) Main factorial plan (F1,F2) of the PCA of milk composition data showing the distribution of the dromedary (in red) and Bactrian (in green) samples; (**b**) correlations circle of the different milk components, pH, SCC and FDP with the first two factors of the PCA.

In the main factorial plan (F1, F2), the two species appeared clearly separated (Figure 1a). According to the correlation circle (Figure 1b), it was possible to observe that the Bactrian samples (in green) were correlated to high fat, high protein, while dromedary samples (in red) were correlated to most of the trace minerals and potassium. Calcium, phosphorus and zinc were less clearly correlated to one species.

### 3.4. Relationships Between Species, Body Measurements and Milk Production

Almost all the body measurements differed between dromedary and Bactrian, except the body length. Also, the mammary vein diameter and the width of the udder were not significantly different (Table 4). Moreover, although on average, milk production was higher in dromedary than in Bactrian, the difference was not significant.

According to the correlation matrix involving both species, the milk production was not associated with any of the body measurements including those regarding the udder width, the teat length or the mammary vein diameter (Table 5). On the other hand, due to the allometric development, most of the body measurements were correlated with each other.

However, according to the analyses of correlations within each species, milk production was significantly and positively associated with the heart girth in the dromedary only (r = 0.529; *p* = 0.014).

### 3.5. Relationships Between Phenotypes, Milk Production and Milk Composition

**Figure 2:**
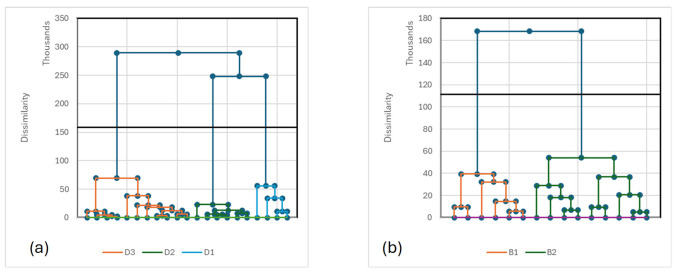
Dendrograms issued from the automatic classifications of body measurements of the dromedaries (**a**) and of the Bactrians (**b**).

After automatic classification of the body measurements profiles of the dromedary and Bactrian camels, three types of dromedaries and 2 types of Bactrian camels were identified (Figure 2).

The three phenotypes of dromedary camels and the two phenotypes of Bactrian camels were described as follows:

- Type D1 (*n* = 4) characterized by long neck, medium height and narrow chest (heart girth) with a low development of the udder and the lowest milk production,
- Type D2 (*n* = 6) characterized by its height and length, wide chest and udder with long teats as well as large mammary vein linked to highest milk production,
- Type D3 (*n* = 11) was the shorter dromedary (LN, HW, TC, FL), but mostly intermediate between the two former phenotypes for the other criteria, especially regarding the udder development and milk production.
- Type B1 (*n* = 6) had a smaller size but with a larger chest and lower milk production,
- Type B2 (*n* = 9), at the reverse had the larger size in almost all criteria (Table 6).

**Figure 3:**
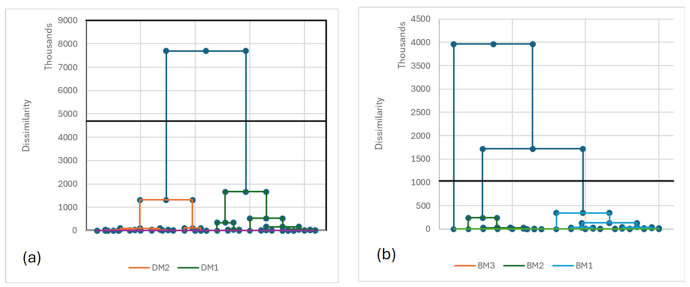
Dendrograms issued from the automatic classifications of milk composition of the dromedaries (**a**) and of the Bactrians (**b**).

A similar approach was achieved for milk composition data, leading to the identification of two milk profiles for dromedaries and three for Bactrians (Figure 3a,b), namely DM1, DM2 and BM1–BM3, respectively. It was noticeable that the group BM3 was composed of one animal only.

The two types of dromedary milk profiles and the three Bactrian milk profiles were described as follows:

- Type DM1 (*n* = 10) characterized by fatty milk poor in minerals except iron and with the lowest milk production,
- Type DM2 (*n* = 11) at reverse was low-fat milk (3.4%), rich in almost all minerals, and with the highest milk production.
- Type BM1 (*n* = 8) was milk samples with a high concentration of main minerals (Ca, K, Mg, P) with relatively high fat content and intermediate milk production
- Type BM2 (*n* = 6) was milk profiles relatively poor in fat and protein but with intermediate mineral contents and the highest milk production among the Bactrian
- Type BM3 (*n* = 1) was a Bactrian milk sample high in fat and protein, but very low in all types of minerals, and linked to low milk production (Table 7).

The contingency tables crossing for each species, the phenotypes and the milk profiles, analyzed by Chi^2^ test, gave no relationships for both dromedary and Bactrian, significant the absence of a link between phenotypes and the composition or volume of milk produced.

## 4. Discussion

In Kazakhstan, the two species of large camelids cohabitate sometimes in the same farm, i.e., exactly in the same feeding and management environment. Unfortunately, in this case, crossbreeding between the two species is commonplace, making it difficult to analyze interspecific differences. As for “purebred” farms, or at least those with this official status certified by the Ministry of Agriculture, they are generally located in distinct regions. *Aruana* camel farms are more frequent in the Turkestan region, while pure Bactrian camel farms are more common around the Caspian Sea. Thus, the differences observed in milk composition could be linked to both the breed and farm effect, although precautions (same lactation stage, comparable parity distribution, similar feeding management, same milking practice) were taken to limit the impact of farm management.

### 4.1. Camel Phenotypes

The clear morphometric differences between *Camelus dromedarius* and *Camelus bactrianus* indicate that gross external morphology alone may not fully explain differences in lactation performance, as also noted in recent studies, where body size is a weak predictor of milk yield, whereas physiological and genetic factors play a central role [16,23]. Although *C. dromedarius* generally shows higher milk yield potential than *C. bactrianus*, the data obtained showed that this difference was not statistically significant, at least in the conditions of our two sampled farms. Such inter- and intra-species variability has been documented under diverse ecological conditions, reflecting the influence of management, feeding, and genetic background [24,25,26]. However, the lack of a relationship between udder morphometry and milk production is surprising. Indeed, in Saudi Arabia, a significant correlation was found for dromedary between some udder morphology traits, notably, mammary vein diameter and dairy yield [27]. A similar observation was made in Sudan [28] and Pakistan [29].

The only significant correlation for dromedary between body measurements and milk production in our study included the heart girth. A similar observation was reported in the dromedary camel from Ethiopia [30]. Heart girth is a good predictor of the total weight of a camel [31] and linked to the body reserves of the animal [32]. Thus, the relationship with milk production could suggest that greater body reserves and overall condition may support enhanced lactational performance under extensive pastoral systems.

The multivariate analysis identified three distinct dromedary and two Bactrian phenotypes, reflecting substantial intraspecific morphological variation within farms. Dromedary Type D1, characterized by long neck (LN), medium height at withers (HW), narrow chest (heart girth), low udder development, and the lowest milk production, corresponds to leaner body conformations that are often prioritized for mobility and resilience under harsh extensive systems rather than high dairy output. Similar patterns have been observed in morphometric analyses where tighter body conformation was associated with lower production traits [33]. Type D2 showed greater height (HW), longer body length, wider chest, well-developed udder, longer teats, and larger mammary vein diameter, corresponding to the highest milk production among the dromedary phenotypes. This association is consistent with evidence from other camel populations indicating that udder and chest dimensions are among the body measurements most closely related to milk yield [23]. In that study, udder circumference and abdominal girth showed moderate correlations with daily milk production, suggesting that conformational traits related to udder capacity and body reserve can reflect productive potential when other environmental and management factors are similar. Type D3, which was shorter in terms of neck length (LN), height at withers (HW), thigh circumference (TC), and foreleg length (FL), yet intermediate between D1 and D2 in udder development and milk yield, highlights the continuum of body conformation within dromedary populations rather than strictly discrete categories. This supports the view that phenotypic variation in camels is continuous and shaped by both genetic background and environmental adaptation, making single trait-based classification less reliable for productivity prediction. Among Bactrian camels, Type B1 was smaller overall but had a larger chest and lower milk yield, whereas Type B2 displayed a larger body size in most measurements. Such intraspecific variability has parallels with reported morphological and productive differences among Eurasian Bactrian camel populations, where larger and more robust individuals tended to show higher milk yield under controlled feeding conditions [4].

Importantly, Chi-square analyses revealed no significant association between morphotype classification and milk profiles in either species. This indicates that external morphometric classification alone does not reliably predict quantitative or compositional milk traits, reinforcing that morphological categories capture phenotypic diversity but not necessarily functional dairy performance. Similar conclusions have been drawn in camel research, where studies using test-day milk records and morphometric measures found only moderate correlations, and many morphometric traits did not consistently explain variation in milk yield across individuals or breeds [23].

Taken together, these findings underscore the complexity of lactation biology in camels. While body conformation, especially chest and udder dimensions, can contribute to explaining some variation in milk production, productive traits are influenced by a combination of genetic, physiological, and environmental factors that morphometric classification alone cannot capture fully. Genomic studies have begun to dissect the molecular basis of milk production traits, identifying candidate genes and pathways that differentiate low- and high-yield animals [34], pointing to the potential for integrating molecular tools with phenotypic data in future selection strategies. These findings are consistent with modern camel research showing that lactation performance is influenced by a complex interplay of genetics, physiological condition, environment, and management practices, rather than body measurements alone [24,35,36]. For example, transcriptomic studies in Bactrian camels have identified genetic pathways affecting milk production independently of external morphology [34]. Seasonal and nutritional factors further modulate milk yield and composition, reinforcing that single-point morphometric assessments may not capture the dynamic lactation potential [36,37].

### 4.2. Milk Composition

To avoid factors other than species influencing variation in the milk composition, milk samples were collected at the same stage of lactation and therefore during the same season of the same year. Furthermore, the parity distribution was comparable in both populations. Finally, the milk collection conditions were identical in both cases. However, despite a comparable pastoral environment, differences in the respective diet cannot be ruled out [38].

The comparative analysis of milk from *Aruana* dromedaries and Kazakh Bactrian camels reveals a significant divergence in nutrient partitioning that underscores the biological specialization of these two species within the Central Asian ecosystem. Our findings demonstrate that the Kazakh Bactrian camel produces a significantly more concentrated milk, characterized by higher fat content and protein concentration compared to that observed in *Aruana* dromedaries. Such a difference was already reported in the few references that compared both species in a similar environment [6]. This richness in macronutrients aligns with the evolutionary requirement of the Bactrian camel to provide a higher energy density to its offspring in the extreme cold deserts of the Caspian region, where metabolic maintenance costs are substantially higher than in the hot arid zones typical of dromedary evolution. The protein fraction emerged as the most stable interspecies marker in our study, confirming that nitrogenous compounds are less susceptible to the environmental fluctuations that often confound lipid analysis. In addition, throughout lactation, the variability of protein concentration appears more stable than fat content in both dromedary [39,40] and Bactrian camel [9,41]. According to literature, the Kazakh Bactrian’s protein levels are superior to the average reported for dromedaries (3.2%) in most Middle Eastern studies, while remaining comparable to the high-solid profiles of Mongolian Bactrians, which often exceed 3.8% protein during peak production [6,38].

While the macronutrient analysis favors the Bactrian species, the mineral and trace element profiles present a more complex “biological signature” that favors the *Aruana* dromedary in specific metrics. Indeed, in our observations, *Aruana* milk was significantly richer in magnesium and manganese compared to the Kazakh Bactrian. This divergence in magnesium concentration, which was highly significant, likely reflects the opportunistic grazing behavior of dromedaries in the southern Turkistan region (Otyrar district), where they browse on halophytic vegetation, especially *Haloxylon aphyllum* (“Saksaoul”), a *Chenopodiaceae* widely present in the region, highly appreciated by the camel, and naturally rich in these ions [42]. Conversely, macro-minerals like calcium and phosphorus remained relatively stable across both species in our Kazakhstani samples, with calcium levels (3557–3851 µg/mL) being remarkably higher than the 1100–1300 µg/mL typically reported for dromedaries in the Arabian Peninsula [43]. This suggests that the Kazakh environment—potentially through soil mineralization or unique forage types—provides an abundant baseline for skeletal-building blocks, regardless of species. The lack of significant variation based on lactation rank (parity) during the 5th month of our study indicates that camel milk reaches a period of “compositional equilibrium” in mid-lactation, simplifying the technical requirements for national standardization.

From a regulation perspective, the ability to biochemically identify “Pure *Aruana*” versus “Pure Kazakh Bactrian” milk is essential to prevent food fraud, specifically the dilution of high-value Bactrian milk with more abundant dromedary milk [44]. Furthermore, our analysis of SCC and pH confirmed that the observed differences were biological and not artifacts of subclinical mastitis or hygienic failure, as all samples remained within healthy physiological ranges [45].

### 4.3. Milk Standard

International regulatory approaches to milk and dairy products are generally guided by the recommendations of the Codex Alimentarius Commission, which provides general principles for food safety and quality. In addition, the technical regulations of the Eurasian Economic Union—TR CU 021/2011 and TR CU 033/2013—serve as fundamental regulatory frameworks for milk safety and quality in several countries across Eurasia and Central Asia where these regulations or similar systems are applied [46,47]. These regulations define basic safety requirements, including microbiological criteria and permissible levels of contaminants such as toxic elements, mycotoxins, pesticides, antibiotics, and radionuclides. However, most international regulatory documents primarily address milk obtained from conventional dairy species and provide limited guidance on camel milk, which differs significantly in its physicochemical composition, thermal stability, and microbiological characteristics [48,49]. For this reason, several countries have developed specific national standards regulating pasteurized camel milk: UAE.S/GSO 1970:2010 in the United Arab Emirates, GSO 1970:2021 adopted by the Gulf Cooperation Council, DKS 2026:2016 in Kenya, and NM 08.4.300:2016 in Morocco [50,51,52,53]. These standards generally establish physicochemical parameters, microbiological requirements, and safety limits for contaminants in camel milk. Some of them primarily address milk from the dromedary camel (*Camelus dromedarius*), while others cover both dromedary and Bactrian camels.

The absence of unified technical requirements and quality criteria for pasteurized camel milk contributes to variability in product quality and may reduce the level of consumer protection in the camel dairy sector. Therefore, the development of scientifically justified standards defining the quality, safety, and authenticity of pasteurized camel milk is essential. Such standards are intended to establish unified terminology, define general product characteristics, determine key physicochemical and microbiological indicators, and specify requirements for packaging, labeling, transportation, and storage. In the context of growing global interest in camel milk as a functional and nutritionally valuable product, the establishment of standardized quality parameters based on reliable scientific data—such as milk composition and productivity of different camel species—is particularly important [54]. Given the limited harmonization of standards and the biological differences between camel species, scientific data on milk composition and productivity are essential for the development of evidence-based regulatory frameworks. In this context, the present study on the milk composition and productivity of *Aruana* dromedary and Kazakh Bactrian camels (both being pure breeds) provides an important scientific basis for the establishment of a national standard for camel milk, especially in a context where the crossbreeding between the two species is common and not properly managed everywhere. Such a standard should ensure two key principles: the safety of the product for consumers and the conformity of marketed camel milk to its declared composition and nutritional value, thereby preventing adulteration and ensuring transparency in the camel milk market [55].

Such results align with the 2025 approval of the first international Codex standard for pasteurized camel milk, which emphasizes the need for species-specific compositional criteria [56]. By formalizing the superior energy density of the Kazakh Bactrian and the unique mineral profile of the *Aruana*, Kazakhstan can create a standard that not only protects consumer rights but also supports the economic deep-processing of traditional products like *shubat* (traditional fermented milk) and high-value exports like “*Saubota*”™ powder.

## 5. Conclusions

The most important differences between dromedary and Bactrian milk, the second one being richer in fat and protein, lower in magnesium and manganese, but less abundant in quantity, were confirmed by the present study despite the limitations in the methodology used due to a limited number of involved farms. While morphometric descriptors like NL, HW, TC, FL, udder width, and heart girth provide useful phenotypic information, selection for enhanced milk production should integrate physiological, genetic, and environmental indicators. The significant association between heart girth and milk yield in dromedaries suggests that composite body condition indices may be valuable selection tools, but robust breeding programs require longitudinal production records and molecular markers to optimize dairy potential in both *C. dromedarius* and *C. bactrianus*.

In addition, the establishment of a standard for both breeds in the case of dromedary and Bactrian milk in a country such as Kazakhstan, where the two species have been cohabiting for centuries, should be carefully supported by an investigation into pure breeds. However, it is still necessary to validate these initial results on a larger number of farms with purebred animals. Moreover, the present official methodology to control the status of “pure-breed” animals in the country should be improved by the introduction of genomic tools, which represents a unique opportunity to detect “Bactrian genes” in the dromedary genome and vice versa.

## Figures and Tables

**Table 1:** Comparative composition (mean and SD), pH, SCC and FDP of Kazakh *Aruana* dromedary and Kazakh Bactrian milk at the fifth month of lactation (mineral units were in µg/mL).

Parameters	Species		*p*
	Dromedary	Bactrian	
Fat (%)	3.59 ± 1.22 ^b^	5.27 ± 0.87 ^a^	**0.002**
Protein (%)	3.18 ± 0.28 ^b^	3.97 ± 0.29 ^a^	**<0.0001**
Ca	3557.41 ± 721.2 ^a^	3851.55 ± 657.9 ^a^	0.357
K	181.15 ± 30.18 ^a^	156.82 ± 36.21 ^a^	0.118
Mg	1357.07 ± 246.2 ^a^	929.40 ± 166.8 ^b^	**<0.0001**
P	360.83 ± 164.36 ^a^	398.87 ± 81.88 ^a^	0.814
Fe	2.31 ± 3.51 ^a^	1.45 ± 0.42 ^a^	0.628
Cu	0.20 ± 0.2 ^a^	0.11 ± 0.07 ^a^	0.254
Zn	6.89 ± 1.86 ^b^	8.61 ± 2.38 ^a^	0.082
Mn	0.10 ± 0.05 ^a^	0.03 ± 0.01 ^b^	**<0.0001**
Se	0.04 ± 0.01 ^a^	0.04 ± 0.01 ^a^	0.937
pH	6.59 ± 0.11 ^a^	6.48 ± 0.08 ^b^	**0.027**
SCC (×1000)	413.65 ± 404.87 ^a^	298.82 ± 331.15 ^a^	0.184
FPD (°C)	−0.62 ± 0.02 ^a^	−0.64 ± 0.08 ^a^	0.446

^a,b^ Each parameter for each species with different subscripts is significantly different at *p* < 0.05. Significant probabilities are in bold character. No significant difference was observed for SCC and freezing point, but a slight statistical difference (*p* < 0.05) occurred for the pH, although on a biological level, the values are within the expected standards.

**Table 2:** Mean values of the different camel milk components (fat, protein and minerals) according to the lactation (all mineral units were µg/mL).

Parameters	Dromedary				Bactrian			
	L1	L2	L3	L4	L1	L2	L3	L4
Fat (%)	3.71	2.76	3.71	4.30	4.68	6.16	4.60	5.18
Protein (%)	3.03	3.17	3.41	3.11	4.01	3.77	3.93	4.24
Ca	3434.08	3927.92	3310.94	3387.65	4461.72	3935.12	3833.26	3594.68
K	172.82	199.10	167.05	178.57	173.71	165.09	172.52	133.00
Mg	1414.10	1420.67	1260.50	1349.62	892.08	977.20	953.21	859.25
P	308.18	358.99	311.92	425.15	489.30	435.40	394.17	358.23
Fe	2.12	1.27	3.81	2.00	1.51	1.64	1.57	1.07
Cu	0.20	0.14	0.11	0.34	0.11	0.12	0.14	0.07
Zn	8.80	6.75	6.09	6.71	6.53	10.11	8.88	6.94
Mn	0.10	0.10	0.09	0.12	0.03	0.04	0.04	0.03
Se	0.04	0.04	0.04	0.04	0.04	0.04	0.04	0.04

**Table 3:** Synthesis of the selected parameters in the ascending stepwise model with their discriminating probability (test F and lambda de Wilks).

Steps	Variables	F	Pr > F	λ de Wilks	Pr < λ
1	protein			0.338	<0.0001
2	protein/Mn	14.093	0.001	0.237	<0.0001
3	protein/Zn/Mn	11.974	0.002	0.172	<0.0001
4	protein/Mg/Zn/Mn	4.912	0.034	0.149	<0.0001
5	protein/Ca/Mg/Zn/Mn	6.648	0.015	0.122	<0.0001

**Table 4:** Comparative body measurements and milk production of Kazakh *Aruana* dromedary and Kazakh Bactrian in two pure-breed farms.

Measurements (mm)	Species		*p*
	Dromedary	Bactrian	
HL	479.0 ± 25.7 ^a^	534.7 ± 28.3 ^b^	**0.001**
NL	1146.2 ± 107.9 ^a^	1044.0 ± 59.6 ^b^	**0.002**
NC	796.7 ± 32.9 ^b^	990.0 ± 59.8 ^a^	**<0.0001**
HW	1880.0 ± 112.7 ^a^	1975.3 ± 82.2 ^b^	**0.009**
HG	1846.2 ± 94.5 ^b^	2360.0 ± 82.5 ^a^	**<0.0001**
TC	815.9 ± 49.2 ^b^	904.5 ± 39.0 ^a^	**<0.0001**
TL	54.3 ± 14.5 ^a^	20.3 ± 8.5 ^b^	**<0.0001**
UW	267.6 ± 25.9 ^a^	278.7 ± 51.5 ^a^	0.403
MVD	35.3 ± 56.8 ^a^	21.7 ± 6.8 ^a^	0.119
FL	1274.1 ± 265.1 ^b^	1570.0 ± 52.8 ^a^	**<0.0001**
BL	1504.5 ± 314.6 ^a^	1588.6 ± 59.6 ^a^	0.257
MP (mL/24 h)	1689.8 ± 707.5 ^a^	1297.8 ± 431.2 ^a^	0.065

^a,b^ Each parameter for each species with different subscripts is significantly different at *p* < 0.05. Significant probabilities are in bold character.

**Table 5:** Correlation matrix of the body measurements involving pure *Aruana* dromedaries and Kazakh Bactrians.

Measur.	LH	LN	NC	HW	HG	TC	TL	UW	MVD	FL	BL	MP
LH	**1**	**−0.371**	**0.740**	**0.640**	**0.816**	**0.708**	**−0.615**	0.053	−0.053	**0.732**	**0.517**	−0.036
LN	**−0.371**	**1**	**−0.352**	0.083	**−0.487**	**−0.349**	**0.402**	−0.174	0.080	−0.323	−0.201	0.108
NC	**0.740**	**−0.352**	**1**	**0.439**	**0.896**	**0.713**	**−0.781**	0.212	−0.200	**0.864**	0.266	−0.221
HW	**0.640**	0.083	**0.439**	**1**	**0.582**	**0.599**	−0.299	0.105	0.014	**0.577**	**0.386**	0.015
HG	**0.816**	**−0.487**	**0.896**	**0.582**	**1**	**0.706**	**−0.813**	0.235	−0.118	**0.913**	**0.339**	−0.151
TC	**0.708**	**−0.349**	**0.713**	**0.599**	**0.706**	**1**	**−0.559**	0.009	−0.174	**0.679**	**0.410**	−0.202
TL	**−0.615**	**0.402**	**−0.781**	−0.299	**−0.813**	**−0.559**	**1**	0.064	**0.456**	**−0.814**	−0.026	0.188
UW	0.053	−0.174	0.212	0.105	0.235	0.009	0.064	**1**	**0.538**	0.075	0.162	0.095
MVD	−0.053	0.080	−0.200	0.014	−0.118	−0.174	**0.456**	**0.538**	**1**	**−0.339**	0.190	0.176
FL	**0.732**	−0.323	**0.864**	**0.577**	**0.913**	**0.679**	**−0.814**	0.075	**−0.339**	**1**	0.247	−0.174
BL	**0.517**	−0.201	0.266	**0.386**	**0.339**	**0.410**	−0.026	0.162	0.190	0.247	**1**	0.032
MP	−0.036	0.108	−0.221	0.015	−0.151	−0.202	0.188	0.095	0.176	−0.174	0.032	**1**

The significant correlations at *p* < 0.05 are in bold.

**Table 6:** Barycentres of the different types of dromedaries (D1–D3) and Bactrians (B1, B2) identified by automatic classification of their body measurements (the milk production—MP-was included as supplementary variable).

Types	LH	LN	NC	HW	HG	TC	TL	UW	MVD	FL	BL	MP
Type D1	465.0	1337.5	820.0	1890.0	1750.0	806.5	50.0	252.5	22.5	1342.5	1520.0	1439.3
Type D2	500.0	1141.7	811.7	2015.0	1938.3	873.8	63.7	268.2	27.7	1378.0	1600.0	1951.7
Type D3	472.7	1080.0	780.0	1802.7	1830.9	787.5	55.7	272.8	24.5	1307.2	1566.3	1638.0
Type B1	516.7	1005.0	955.0	1908.3	2273.3	916.0	18.7	266.7	21.8	1533.3	1576.7	1098.7
Type B2	546.7	1070.0	1013.3	2020.0	2417.8	897.4	21.4	286.7	21.6	1594.4	1596.6	1430.6

**Table 7:** Barycentres of the different types of dromedary (D1, D2) and Bactrian (B1–B3) milk profiles identified by automatic classification of their milk components (the milk production—MP-was included as a supplementary variable). Fat and Prot were in %, minerals in µg/mL and MP in mL.

Milk Profiles	Fat	Prot	Ca	K	Mg	P	Fe	Cu	Zn	Mn	Se	MP
DM1	3.9	3.2	2920.4	157.7	1156.4	348.5	3.4	0.2	6.2	0.09	0.04	1234.7
DM2	3.4	3.2	4073.7	201.5	1527.1	375.7	1.4	0.2	7.3	0.11	0.04	1811.6
BM1	5.5	4.0	4259.6	163.4	1006.0	452.8	1.3	0.11	8.6	0.04	0.04	1446.1
BM2	5.0	3.9	3564.4	161.7	898.0	370.3	1.7	0.12	9.0	0.04	0.04	1641.3
BM3	5.7	4.3	1980.4	76.8	451.1	189.7	0.6	0.07	4.3	0.02	0.02	1260.0

## Data Availability

The data supporting the findings of this study are included in the article. Further information is available from the corresponding author upon reasonable request.

## Abbreviations

The following abbreviations are used in this manuscript:

HL	the head length
NL	the neck length
NC	the neck circumference
HW	the height at withers
HG	the heart girth
TC	the thigh circumference
TL	the teat length
UW	the udder width
MWD	the mammary vein diameter
FL	the foreleg length
BL	the body length
MP	the milk production
SCC	somatic cell count
FPD	the freezing point
ICP-OES	inductively coupled plasma optical emission spectrometer
DFA	the discriminant factorial analysis
PCA	the principal component analysis
AHC	the automatic hierarchical classification
UAE	United Arab Emirates
GSO	Gulf Cooperation Council
